# The Cerebellum on Cocaine

**DOI:** 10.3389/fnsys.2020.586574

**Published:** 2020-10-21

**Authors:** Marta Miquel, Isis Gil-Miravet, Julian Guarque-Chabrera

**Affiliations:** Área de Psicobiología, Universitat Jaume I, Castellón de la Plana, Spain

**Keywords:** cerebellum, drug addiction, prefrontal cortex, ventral tegmental area, striatum, goal-directed behavior, habit

## Abstract

The traditional cerebellum’s role has been linked to the high computational demands for sensorimotor control. However, several findings have pointed to its involvement in executive and emotional functions in the last decades. First in 2009 and then, in 2016, we raised why we should consider the cerebellum when thinking about drug addiction. A decade later, mounting evidence strongly suggests the cerebellar involvement in this disorder. Nevertheless, direct evidence is still partial and related mainly to drug-induced reward memory, but recent results about cerebellar functions may provide new insights into its role in addiction. The present review does not intend to be a compelling revision on available findings, as we did in the two previous reviews. This minireview focuses on specific findings of the cerebellum’s role in drug-related reward memories and the way ahead for future research. The results discussed here provide grounds for involving the cerebellar cortex’s apical region in regulating behavior driven by drug-cue associations. They also suggest that the cerebellar cortex dysfunction may facilitate drug-induced learning by increasing glutamatergic output from the deep cerebellar nucleus (DCN) to the ventral tegmental area (VTA) and neural activity in its projecting areas.

## Introduction

It is remarkable that during evolution there has been a coordinated scaling in the number of cortical and cerebellar neurons across species (Herculano-Houzel, 2010). However, in terms of the number of neurons contained in the brain mass, the cerebellum is the winner with 80% of neurons, the majority granular cells, packed in 10% of brain volume (Azevedo et al., 2009). It is still uncertain why so many neurons are needed for cerebellar functions as silencing 75% of granule cells does not impact motor adaptation and performance (Galliano et al., 2013). It has been suggested as one possible explanation that the huge number of granule cells enable to make sparse coding available to increase memory storage capacity in the cerebellum (Schweighofer et al., 2001). In evolutionary terms, it would have allowed great apes to improve the learning of motor skills, tool-making and in the end, verbal communication (Barton, 2012).

**FIGURE 1:**
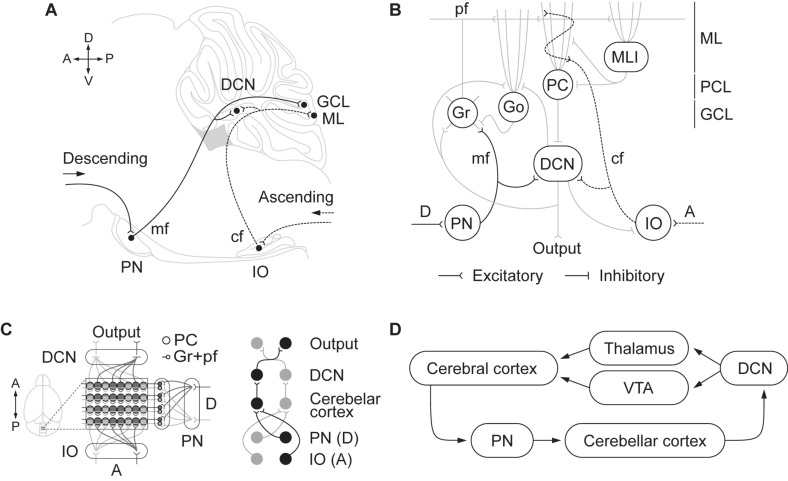
**(A)** Descending and ascending afferents to the cerebellum, cerebro-pontinocerebellar and spino-olivocerebellar pathways, respectively. Descending cortical efferents arrive to the pontine nuclei (PN) that provides mossy fiber (mf) projections to the cerebellar cortex ending in the granule cell layer (GCL). The ascending pathway from to the inferior olive (IO) projects climbing fibers (cf) to the cerebellar cortex contacting Purkinje cell (PC) dendrites in the molecular layer (ML). Both pathways leave collaterals in the deep cerebellar nuclei (DCN) on their direction to the cerebellar cortex. **(B)** Schematic representation of the cerebellar circuitry. The descending pathway through mf contacts granule (Gr) and Golgi (Go) cells in the GCL and leaves collaterals in the DCN. Gr send their ascending axon to the ML that bifurcates forming the parallel fibers (pf). In the ML, pf make contact with the dendrites of Go, PC, and molecular layer interneurons (MLI). The ascending pathway through cf contacts PC dendrites. PC sends inhibitory projections to the DCN that only sends output signal when PC are inhibited by MLI. DCN not only send the main cerebellar output but also send inhibitory feedback to IO and Go, and excitatory feedback to Gr and Go. The cerebellar circuit is organized as a feedforward excitatory network with inhibitory loops. **(C)** Modular organization of the cerebellum. The anatomical organization of the cerebellum is distributed in longitudinal modules, where PC are organized perpendicular to the cortical folds. Moreover, differential microzones can be observed forming striped zones of PC. Each module is organized by parasagittal bands of PC and the cf emerging from the contralateral IO that contact them. Mf projecting to a certain group of PC through the GCL also contact with the same DCN those PC project to. In that manner there is somatotopy between the deseeding and ascending pathways, the PN and IO regions, the cerebellar cortical zones where mf and cf terminate in, and the specific DCN region where PC project to. **(D)** Cortico-cerebellar loops.

It is now clear that reciprocal connectivity between the cerebellum and the rest of the brain is crucial to understanding cerebellar function (Galliano and De Zeeuw, 2014; Lackey et al., 2018). A key principle of the anatomical organization in the cerebellum is the segmentation of inputs and outputs in several longitudinal oriented modules (Figure 1). Each module includes descending and ascending afferents from subdivisions in the pontine nuclei and inferior olive, respectively. These inputs reach specific areas in the cerebellar cortex as well as regions of deep cerebellar and vestibular nuclei to which Purkinje cells project their axons (see for a recent review, Watson and Apps, 2019). Cerebellar modules integrate information from differentiated brain areas with incoming peripheral signals (Figure 1). Accordingly, sensorimotor coordination depends on the convergence at a cellular level of somatosensory and motor cortical inputs into specific regions of the cerebellar cortex that, in turn, send ascending projections to the motor cortex through differentiated thalamic areas (Proville et al., 2014). Moreover, the functional loop formed by the anterior lateral motor cortex and fastigial nucleus in the cerebellum is required to maintain the representation of the information in the frontal cortex during motor planning, as Gao et al. (2018) recently demonstrated.

**FIGURE 2:**
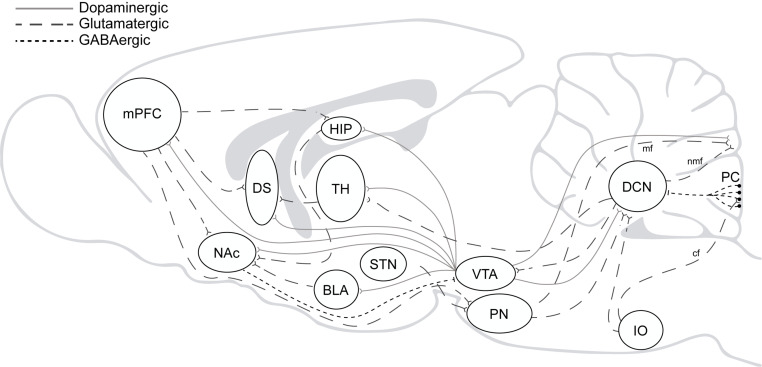
The addiction circuitry. mPFC, medial prefrontal cortex; DS, dorsal striatum; NAc, nucleus accumbens; HIP, hippocampus; BLA, basolateral amygdala; TH, thalamus; STN, subthalamic nucleus; VTA, ventral tegmental area; PN, pontine nucleus; IO, inferior olive; DCN, deep cerebellar nucleus; PC, purkinje cells; mf, mossy fibers; nmf, nuclear mossy fibers; cf, climbing fibers. Direct and indirect reciprocal connectivity between the cerebellum and other brain regions as the VTA, amygdala, basal ganglia, and mPFC.

Beyond the well-accepted role of the cerebellum in the high computational demands for sensorimotor control, several findings have pointed to its involvement in executive and emotional functions in the last decades (Schmahmann and Sherman, 1998; Blackwood et al., 2004; Bastian, 2006; Callu et al., 2007; Cardoso et al., 2014; Nguyen et al., 2017; Deverett et al., 2019; Hull, 2020). Direct and indirect reciprocal connectivity between the cerebellum and other brain regions as the amygdala, basal ganglia, and mPFC can explain cerebellar roles in fear memory, cognitive flexibility, behavioral control, and goal-directed behavior (Sacchetti et al., 2002; Bostan et al., 2013; Wagner et al., 2017; Xiao et al., 2018; Schmahmann, 2019). Likewise, it can also explain why cerebellar dysfunction is associated with neuropsychiatric disorders in which impairment of behavioral and cognitive inhibitory control is a central part of the disease pattern as to happen in autism, obsessive-compulsive (OCD), attention deficit/hyperactivity (ADHD) and addiction (Miquel et al., 2019; Figure 2).

Accordingly, in 2009 and then in 2016, we raised the question to why we should consider the cerebellum when thinking about drug addiction and drew attention to the cerebellum’s roles in several of the brain functions affected in drug addicts. A decade later, mounting evidence strongly suggest the cerebellar involvement in this disorder. It is worth noting that the cerebellum connects bidirectionally to functional loops involved in drug addiction. In particular, the cerebellum modulates cortical influences on striatal activity (Chen et al., 2014; Watson et al., 2014) and exerts an indirect (Forster and Blaha, 2003; Rogers et al., 2011) but also a direct control over VTA dopaminergic neurons (Watabe-Uchida et al., 2012; Carta et al., 2019; Gil-Miravet et al., 2019b; Figure 2). Nevertheless, evidence is still partial and related mainly to drug-induced reward memory, but recent results may provide with a wider perspective of cerebellar roles in drug addiction. For the purpose of this minireview, we will focus on specific findings about the role of the cerebellum in cocaine-induced learning, suggesting the way forward for future research.

## A Brief Historical View of Addictive Drug Effects on Cerebellar Function

First evidences of drug effects on the cerebellum came from the toxicology of alcohol and cannabis. For many years, it was thought that the main role of the cerebellum was limited to tolerance to motor incoordination and some of the withdrawal symptoms induced by drug misuse (Killicorn, 1955; Decker et al., 1959; Allsop and Turner, 1966; Ho et al., 1972; Dewey et al., 1973; Vivian et al., 1973; Rogers et al., 1980; Liljequist and Tabakoff, 1985; Rodríguez de Fonseca et al., 1994; Blednov et al., 2017). It is undeniable that alcohol and cannabis induce molecular changes in the cerebellum that correlate with homeostatic adaptations. Reduction of inhibitory binding sites and downstream pathways (Dewey et al., 1973; Liljequist and Tabakoff, 1985; Rodríguez de Fonseca et al., 1994; Casu et al., 2005) or decreases in cerebellar responses to these drugs (Volkow et al., 1993; Blednov et al., 2017) are both examples of homeostatic adaptations with chronic drug use. Nevertheless, drug impact on the cerebellum goes beyond homeostatic alterations and entails similar plasticity changes that have been described in the basal ganglia and mPFC to underly incentive sensitization (Couceyro et al., 1994; Klitenick et al., 1995; Bhargava and Kumar, 1999; Fumagalli et al., 2007; Palomino et al., 2014) and cocaine-induced associative memory (Carbo-Gas et al., 2014a, b, 2017).

A critical part of aberrant drug-induced plasticity in several brain regions is linked to Brain-Derived Neurotrophic Factor (BDNF) mechanisms (Li and Wolf, 2015). Indeed, relapse risk correlates with serum BDNF levels in abstinent cocaine addicts (Grimm et al., 2003; D’Sa et al., 2011; Corominas-Roso et al., 2015). Likewise, time-dependent increases in BDNF in the mPFC and VTA, but not in the nucleus accumbens (Bobadilla et al., 2019), have been associated with the incubation of craving under cocaine abstinence (Grimm et al., 2003). We have explored the effects of cocaine reinstatement on BDNF-related plasticity in the cerebellum of sensitized mice after short (Vazquez-Sanroman et al., 2015a) and protracted abstinence (Vazquez-Sanroman et al., 2015b). The direction of cerebellar plasticity after reinstatement depends entirely on the length of abstinence. Cocaine reinstatement after short abstinence (1 week) promotes transcriptional activity through the overexpression of exon VI in the cerebellar cortex that resulted in an accumulation of proBDNF to the detriment of matureBDNF isoform. Accordingly, p75NGF receptor levels also increased. ProBDNF expression enhanced in Purkinje neurons and Bergman glia, and was associated with structural changes such as pruning of Purkinje dendritic spines and a reduction in the size and density of their synaptic terminals. Moreover, GluR2 subunits of AMPA receptors in Purkinje neurons appeared internalized (Vazquez-Sanroman et al., 2015a). Therefore, shortly after drug cessation, cocaine reinstatement reduces the inhibitory tonic control of Purkinje neurons on DCN output neurons.

In contrast, cocaine reinstatement after protracted abstinence (1 month) increased mature-BDNF mechanisms in the cerebellar cortex of cocaine sensitized mice. The increase was a post-transcriptional effect plausibly fueled through the enhancement of tissue plasminogen activator (tpA) levels responsible for proBDNF cleavage (Vazquez-Sanroman et al., 2015b). In consequence, we observed higher levels of mature-BDNF isoform and TrkB receptors, as well as increased cellular BDNF expression in Purkinje neurons. BDNF-related plasticity was accompanied this time by dendritic sprouting and a larger size of synaptic boutons in Purkinje neurons. Moreover, GluR2 expression enhanced selectively in the soma and dendrites of these cells in lobules VIII and IX of the vermis, suggesting GluR2 trafficking toward the cell surface (Vazquez-Sanroman et al., 2015b). It has been shown that the mature-BDNF isoform may promote activity-dependent dendritic sprouting and axonal re-modeling in striatal-cortico-limbic neurons through TrkB receptor activation (Kafitz et al., 1999; Kovalchuk et al., 2002). Similar role has been demonstrated for BDNF and TrkB receptors in parallel fiber-Purkinje cell synapses (Lu and Figurov, 1997). Thus, although it remains to be tested, these results suggest that cocaine reinstatement after protracted abstinence would promote enhanced Purkinje inhibitory functions decreasing the cerebellar output. Altogether, these findings indicate a time-dependent regulation of molecular and structural plasticity in Purkinje neurons during withdrawal that might contribute to promote cocaine seeking and relapse.

## Cerebellar Role in Cocaine-Induced Associative Memory

The central role of the cerebellum in associative motor and non-motor learning has been well established (Kim and Thompson, 1997; De Zeeuw and Yeo, 2005; Sacchetti et al., 2005). It has been shown that both electrical (Steinmetz et al., 1989) and optogenetic stimulation (Albergaria et al., 2018) of mossy fiber inputs from the pontine nuclei projecting to the granular cell layer mimic the conditioned stimulus to produce conditioned eyeblink responses. Acquisition and short-term expression of the conditioned motor response depend on the specific regions in the cerebellar cortex (Galliano et al., 2013). However, long-term consolidation seems to occur in the interposed nucleus (Carulli et al., 2020).

In drug addiction, learned reinforcing properties make drug-associated cues strong motivational triggers for drug seeking. The consolidation and persistence of these associative memories (Everitt and Robbins, 2005; Hyman et al., 2006) together with deficits in executive inhibitory control (Koob and Volkow, 2010) result in an elevated risk of relapse even after long periods of protracted abstinence. Long-lasting brain changes that underlie drug-related memories make addiction a chronic disease.

A strong support for a cerebellar role in long-lasting drug-cue associations comes from human and animal research (Miquel et al., 2009, 2016; Moulton et al., 2014; Moreno-Rius and Miquel, 2017). Neuroimaging studies of cue-reactivity have consistently shown cerebellar activations when drug-related cues are presented to drug addicts (Grant et al., 1996; Martin-Sölch et al., 2001; Schneider et al., 2001; Anderson et al., 2006; Smolka et al., 2006; Filbey et al., 2009). The cerebellar activation has been described regardless the sensory modality and the type of drug used. Several findings make unlikely cerebellar activity to be a craving correlate (Moreno-Rius and Miquel, 2017). First, overlapping patterns of cerebellar activation have been described when drug abusers are presented with food- and drug-related cues (Tomasi et al., 2015). Second, the presentation of anger-associated cues also triggers increased activity in the cerebellum (Kilts et al., 2001). Last but not least, a positive correlation between cerebellar activations and craving self-reports is far from being a consistent result (Grant et al., 1996; Bonson et al., 2002; Risinger et al., 2005).

We have been investigating the cerebellum’s contribution to cocaine-cue associative memory during the last decade (Carbo-Gas et al., 2014a, b, 2017; Gil-Miravet et al., 2019a, b). Using an animal model of cocaine-induced odor preference conditioning, we have shown that the conditioned preference for cocaine-associated cues correlates with a selective increase in neural activity (cFos expression) at the apical part of the granular cell layer in the posterior vermis (lobules 8 and 9). The increased activity was observed in granule cells mainly, and in about half of Golgi interneurons (Carbo-Gas et al., 2014a, b, 2017). It has been described that the number of activated granule cells rises as learning progresses (Giovannucci et al., 2017). It is worthy of mention that no activity changes were detected in the cerebellum of pseudo-conditioning animals or those that did not express the conditioned response. These findings rule out the possibility that the cerebellar activation results exclusively from unconditioned stimulating properties of cocaine or from movements performed during the test, since all treated groups received the same drug regimen and no differences were found in motor activity (Carbo-Gas et al., 2017). Furthermore, they suggest that granule cell activation would represent some aspects of the drug-cue associative engram or, at least, use this engram to regulate goal-directed behavior. According to the last suggestion, increased granule cell activity was not seen when mice showing preference were confined in the presence of CS+ (cocaine-related cue), but with no chance of selecting between CS+ and CS− (saline-related cue) (Carbo-Gas et al., 2017). Thus, granule cell activity did not result from the cue presentation merely. It seems to be more related to response selection driven by the reward-predictive cue. In addition, the main cerebellar task during the action selection might involve the generation of intentions to reach the goal as it has been recently suggested (Caligiore et al., 2017). In other words, the cerebellum would contribute to bias behavioral selection toward the context that predicts drug availability (Carbo-Gas et al., 2014b). The internal state (drug abstinence) might modulate these cerebellar predictions, increasing the probability of selecting the drug-associated context when the drug is absent from the body. Nevertheless, although the representation and estimation of the goal value appears to be a role of the cortico-basal ganglia loop (Houk and Wise, 1995; Doya, 2000), we cannot rule out that the cerebellar cortex encodes the reward value of the cocaine associated cue. Granule cell activity in the apical region has been demonstrated to encode reward, the expectation of reward, reward omission and the conditioned response using natural rewards (Wagner et al., 2017). In functional terms, the expected consequence of increasing activity in granule cells is an enhanced inhibitory effect of molecular interneurons over Purkinje activity as parallel fibers contact and can stimulate dendrites of molecular interneurons (Gao et al., 2016; Albergaria et al., 2018). Then, one should expect granule cell activation to facilitate cerebellar output by reducing Purkinje inhibitory control onto DCN neurons. However, we have not been able to demonstrate consistent Purkinje activity changes being linked to cocaine-induced preference memory by using cFos expression analysis (Carbo-Gas et al., 2014a, b).

A significant issue in the addiction field is to explain why drug-induced memories are so persistent and long-lasting. In this respect, we have been interested in the function of perineuronal nets (PNNs) in the cerebellum and their regulation by cocaine-related behavior. PNNs are extracellular matrix lattice cartilage-like structures that wrap the soma and proximal neurites of several neuronal populations, the majority fast-spiking GABAergic interneurons (Grimpe and Silver, 2002; Fawcett et al., 2019). These structures restrict neuronal plasticity and actively contribute to synaptic stabilization in the adult brain (Dityatev and Schachner, 2003; Corvetti and Rossi, 2005). In the cerebellum, only a few neuronal populations are surrounded by PNNs, and unlike other regions, PNN-bearing neurons do not express parvalbumin (Carulli et al., 2006). In a recent study, we have investigated the regulation of cerebellar PNNs by cocaine-induced memory (Carbo-Gas et al., 2017). The acquisition of conditioned preference for cocaine-related cues increases PNN expression surrounding Golgi interneurons in the apical region of the cerebellar cortex. Stronger PNNs correlate with a higher preference for cocaine-related cues. Again, the effect was found in the apical region of the cerebellar cortex and was not reproduced in pseudo-conditioned groups or when conditioned response was not evident. However, PNNs expressed by inhibitory and excitatory neuronal populations in the DCN decreased in all cocaine-treated groups irrespective of the establishment of a conditioned preference. Importantly, previous research has shown that degradation of PNNs in the prelimbic cortex and anterior hypothalamus is able to prevent the formation of drug-related memory (Slaker et al., 2015; Blacktop et al., 2017).

Taken together, our findings indicate that drug-related memory may be represented in the cerebellum through two hallmarks signatures localized in the apical region of the granule cell layer: increased granule cell activity and strong fully condensed PNNs around Golgi interneurons. We have not been able to reproduce either of these effects in ventral regions of the posterior vermis though these regions expressed high granule cell activity and a large number of Golgi cells bearing a PNN (Carbo-Gas et al., 2014a, 2017). These results raise the question of which functional characteristics and connectivity patterns make the apical region relevant to drug associative memory (Gil-Miravet et al., 2019a, b). The apical cerebellar cortex receives mossy fiber inputs from sensorimotor and exteroceptive cortices through specialized regions in the pontine nuclei (Voogd and Ruigrok, 2004). In addition, a prominent excitatory projection of mossy fibers to the apical region of the granule cell layer arises from the DCN and has demonstrated to optimize the conditioned response in motor associative learning (Gao et al., 2016). Finally, granule cell activity in this area is present during unconditioned and conditioned stimuli, as well as during the conditioned response (Giovannucci et al., 2017; Wagner et al., 2017).

In a pursuit of an explanation for the relevance of this cerebellar region, we investigated the effects of a neurotoxic lesion restricted to the apical part of the posterior vermis (lobule 8) on cocaine-related memory (Gil-Miravet et al., 2019a, b). Unexpectedly, the apical lesion dramatically raises the probability of learning/expressing the cocaine-cue Pavlovian association (Gil-Miravet et al., 2019a, b). More important, the cerebellar lesion increased neuronal activity in the mPFC, NAC, and all striatal subdivisions except the ventrolateral striatum, backing the idea of the VTA as a plausible hotspot for a modulatory action of the cerebellum on drug-related memory effects (Carta et al., 2019; Gil-Miravet et al., 2019b). Indeed, using both retrograde and anterograde tracing, we demonstrated that neurons from the apical region of lobule 8 in the vermis reach both the interposed and lateral nuclei (DCN) in the deep cerebellum running laterally to the middle line. In turn, the DCN send direct glutamatergic projections to the contralateral VTA making contacts with dopaminergic and non-dopaminergic neurons. The role of the cerebellum in regulating VTA neuronal activity and conditioned place preference (CPP) has been nicely demonstrated in a recent paper (Carta et al., 2019). Optogenetic stimulation of DCN-VTA glutamatergic projections activated VTA neurons and was sufficient to induce a strong CPP. In agreement, the apical lesion of lobule 8 increased neural activity in the dentate nucleus, likely by reducing the tonic inhibition exerts by Purkinje onto DCN neurons (Gil-Miravet et al., 2019b). We were also interested in the role of the functional loops between the cerebellum and medial prefrontal cortex as well. Our results showed that the deactivation of the infralimbic (IL) but not the prelimbic (PL) cortex during conditioning also facilitates the acquisition of cocaine-induced conditioned preference. Interestingly, simultaneous cerebellum-IL deactivations abolished the facilitative effect of separate deactivations on drug-related learning, indicating a compensatory close IL-cerebellar loop (Gil-Miravet et al., 2019a; Figure 2).

## Model-Free vs. Model-Based Systems in Drug Reward Learning. What Would the Cerebellum’s Role Be?

Reinforcement learning involves two distinct and parallel processes with dissociated neural substrates (Balleine and Dickinson, 1998). During instrumental actions, a flexible and planned goal-directed behavior can compete but also cooperate with the stimulus-response automatic process (habit). When habitual behavior is established, the probability of responding for devalued outcomes increases because the mental representation of the relationship between the stimulus (S) and response (R) does not incorporate information about the current value of the outcome (Adams and Dickinson, 1981; Dayan and Balleine, 2002; Balleine and O’Doherty, 2010). From a theory of decision perspective, these two different systems guide behavior during decision making. A model-based system (goal-directed) that includes an explicit knowledge about the reward context competes with a model-free system (habit) that learn about the reward value and emits habit-like responses with repetition (Lee et al., 2014). The first one operates under uncertainty while the second predominates when outcomes become more predictable. The ability to resolve and monitor the competition between habit and goal-directed processes entails a coordinated work of cortico-basal ganglia-cerebellar loops (Caligiore et al., 2017) and depends on the engagement of executive control (Lee et al., 2014; Watson et al., 2018). It has been proposed that the executive control is under an “arbitrator” that would mediate between planning systems that use predictions of action-outcomes and habitual action selection systems that learn to automate by repeating previously rewarded actions (Lee et al., 2014). The model-free seems to be the default system (Wang et al., 2018). Namely, the arbitrator appears to modulate the activity of brain regions involved in model-free control thereby gating the shift to a flexible behavioral approach. Although it is far to be clear which brain regions may arbitrate this action selection, findings point to inferior medial and lateral prefrontal regions including the rostral cingulate cortex (Lee et al., 2014). Moreover, unquestionable evidence indicates that ventral regions of the basal ganglia receiving dopamine projections from the VTA and SNc are responsible for goal-directed behaviors (de Wit et al., 2009; Balleine and O’Doherty, 2010). Likewise, habit-like responses require the integrity of sensorimotor cortices and dorsolateral regions within the striatum (caudate/putamen) (Yin and Knowlton, 2006; Lee et al., 2014).

Recent findings provide compelling support for the role of the cerebellum in reward-based reinforcement learning and goal-directed behavior (Wagner et al., 2017; Carta et al., 2019; Hull, 2020), but also in model-free learning (Callu et al., 2007; De Bartolo et al., 2009; Liljeholm et al., 2015; Watson et al., 2018). It is clear that cf and Gr cells (Figure 1) generate responses in the cerebellar cortex to events that predict upcoming reward, but they also encode reward omissions (Wagner et al., 2017; Heffley et al., 2018). These signals will enable Purkinje cells to learn responses and establish “forward models” to make effective predictions that regulate behavioral decisions.

One of the most relevant insights into the role of the cerebellum in goal-directed behavior has been provided by the recent study by Carta et al. (2019). In this work, optogenetic stimulation of glutamatergic projections from the DCN to VTA induces consistent short and long-term place preference for the location in which the optogenetic stimulation was applied. Optogenetic stimulation was release upon entry in the reward location. Mice expressing channelrhodopsin2 (ChR2) actively approached this location to obtain the stimulation of the cerebellum-VTA pathway. Moreover, the stimulation was able to increase firing in one third of VTA cells *in vivo*, eliciting excitatory synaptic currents in DA and non-DA neurons. Optogenetic inhibition of cerebellar terminals in the VTA did not induce aversion but prevented the establishment of social preferences. Altogether, these findings reveal that the cerebellum not only is able to encode the goal and the context but also to regulate behavior to reach the goal.

Evidence about the contribution of the cerebellum to habits has come from animal and human research. A bilateral lesion in the interposed nucleus or hemicerebellectomy (Callu et al., 2007; De Bartolo et al., 2009) prevent the establishment of habits with overtraining. In these experiments, animals with the cerebellar alteration maintained the action-outcome features despite overtraining, and expression of an automatic cue-response stage is not created. In humans, the cerebellum and other regions in the sensorimotor network show greater activation when subjects respond to previously devalued outcomes, suggesting their involvement in the expression of S-R habits (Watson et al., 2018; Liljeholm et al., 2015). Indeed, activity in the tail of the caudate/thalamus, the cerebellum and the lingual gyrus predicts insensitivity to outcome devaluation. The greater the activity the higher the probability to respond to devaluated outcomes (Liljeholm et al., 2015). In this case, the cerebellar activity did not result from repetition, since the task did not involve overtraining. On the contrary, it predicted the formation of strong S-R associations.

Drug addiction like other compulsive disorders might result from an excessive dominance of model-free control (Everitt and Robbins, 2005; Lüscher et al., 2020). With extended drug experience, cue-action-reward associations become stronger and drug-associated cues come to be powerful motivational triggers for craving and relapse even after long periods of protracted abstinence. Addictive behavior has been identified as a compulsive habit (Belin et al., 2013). First, drug seeking is automatically triggered by the presence of drug-related cues or their mental representation. Second, behavior becomes insensitive to outcome devaluation (Everitt and Robbins, 2005; Zapata et al., 2010; Belin et al., 2013; Ersche et al., 2016). Compulsive disorders imply a failure in cortical top-down control and over-activity in dorsal regions of the basal ganglia which cause behavioral disinhibition and stereotyped behavioral repetition (Fineberg et al., 2014; Figee et al., 2016).

Structural neuroimage findings also point to a role for the cerebellum in compulsive behavior. The most common finding of patients with compulsive disorders has been decreased gray matter (GM) volume in several regions of the cerebellum and increased basal ganglia-cerebellar connectivity (see Miquel et al., 2019 for a review). Moreover, drug addicts and heavy drug users exhibit a dysfunctional prefrontal-cerebellar pattern in which the cerebellum appears to be overactive during cognitive tasks that should recruit prefrontal resources (Hester and Garavan, 2004; Bolla et al., 2005; Goldstein et al., 2007). Another brain region that has been demonstrated to be crucial for the formation and crystallization of habits is the infralimbic cortex (IL) (Killcross and Coutureau, 2003; Smith and Graybiel, 2013; Barker et al., 2018). Activity in the IL during habit formation is necessary for full habit acquisition. In fact, repeated optogenetic inhibition during overtraining disrupts the formation of habits in rats and mice (Smith and Graybiel, 2013; Barker et al., 2018).

Overall, our recent findings reveal that the cerebellum may modulate cocaine-induced learning, but also neural activity and synaptic stabilization mechanisms in the IL (Gil-Miravet et al., 2019b). Impairment of the posterior vermis increased activity and perineuronal expression in the IL, but only in those rats that expressed the conditioned response of preference for drug associated cues. We have proposed that under stimulation of the cerebellar cortex the prevailing behavioral pattern of drug seeking will be flexible and sensitive to reward devaluation (goal-directed). On the contrary, cerebellar cortex deactivations will encourage the formation of strong cue-drug associations and insensitivity to reward devaluation (habit) (Gil-Miravet et al., 2019b; Miquel et al., 2019).

## Concluding Remarks

Overall, findings point to a modulatory function of the cerebellum on the establishment of drug-induced associative memory through the regulation of VTA activity. Nonetheless, research about the cerebellum’s role in drug-related memory is still in its infancy and there are open questions that remain unanswered. First, it is unknown through which neural pathways drug-related cue and unconditioned signals are conveyed to the cerebellum. Second, it is also ignored how cerebellar cortex encodes the conditioned cue and unconditioned properties of the drug. Third, the role of Purkinje neurons in drug-cue associative memory needs to be further explored. Finally, it is crucial to test the hypotheses including addictive drugs with distinct neuropharmacological actions. It is expected the modulatory role of the cerebellum to affect not only the expression of the conditioned response but also non-motor aspects of drug-induced reward. To address all these questions more precisely cell-specific tools such as DREADDs (designer receptor exclusively activated by designer drugs), optogenetics, electrophysiology and viral tracing will be required.

Our findings also suggest that pathological conditions impairing the cerebellar cortex may increase the likelihood of acquiring drug-induced associative memory and promote drug relapse through disinhibition of VTA and its projecting regions. Moreover, they may explain why both cerebellar disfunction and prefrontal impairment enhance susceptibility to compulsive and impulsive disorders including drug addiction, eating disorders, attention deficit/hyperactivity (ADHD), and obsessive-compulsive disorder (OCD) (Miquel et al., 2019). Accordingly, it has been described that in humans, the impairment of the vermis results in impulsivity and disinhibition (Silveri et al., 1994; Schmahmann and Sherman, 1998; Kim et al., 2013; Tessier et al., 2015), inducing what has been called the Cerebellar Cognitive Affective Syndrome (CAS) (Schmahmann and Sherman, 1998). It would be of the utmost importance to test whether similar cerebellar disfunctions that lead to CAS increase the risk for drug addiction or are sufficient for inducing a compulsive addictive phenotype.

## Author Contributions

Findings discussed in this work were obtained by IG-M and JG-C as a part of their doctoral theses and also critically reviewed the manuscript. MM was responsible for writing and critically edited the review. All the authors approved the final version for publication.

## Conflict of Interest

The authors declare that the research was conducted in the absence of any commercial or financial relationships that could be construed as a potential conflict of interest.
